# Provincial and gridded population projection for China under shared socioeconomic pathways from 2010 to 2100

**DOI:** 10.1038/s41597-020-0421-y

**Published:** 2020-03-09

**Authors:** Yidan Chen, Fang Guo, Jiachen Wang, Wenjia Cai, Can Wang, Kaicun Wang

**Affiliations:** 1 State Key Joint Laboratory of Environment Simulation and Pollution Control (SKLESPC), School of Environment, Tsinghua University, Beijing, 100084 China; 2 Ministry of Education Key Laboratory for Earth System Modeling, and Department of Earth System Science, Tsinghua University, Beijing, 100084 China; 3 Center for Healthy Cities, Institute for China Sustainable Urbanization, Tsinghua University, Beijing, 100084 China; 4 Tsinghua-Rio Tinto Joint Research Center for Resource Energy and Sustainable Development, Tsinghua University, Beijing, 100084 China; 5 College of Global Change and Earth System Science, Beijing Normal University, 19 Xinjiekouwai Street, Haidian, Beijing 100875 China

**Keywords:** Socioeconomic scenarios, Projection and prediction

## Abstract

In response to a growing demand for subnational and spatially explicit data on China’s future population, this study estimates China’s provincial population from 2010 to 2100 by age (0–100+), sex (male and female) and educational levels (illiterate, primary school, junior-high school, senior-high school, college, bachelor’s, and master’s and above) under different shared socioeconomic pathways (SSPs). The provincial projection takes into account fertility promoting policies and population ceiling restrictions of megacities that have been implemented in China in recent years to reduce systematic biases in current studies. The predicted provincial population is allocated to spatially explicit population grids for each year at 30 arc-seconds resolution based on representative concentration pathway (RCP) urban grids and historical population grids. The provincial projection data were validated using population data in 2017 from China’s Provincial Statistical Yearbook, and the accuracy of the population grids in 2015 was evaluated. These data have numerous potential uses and can serve as inputs in climate policy research with requirements for precise administrative or spatial population data in China.

## Abstract (summary)

**Table:** 

Measurement(s)	population
Technology Type(s)	computational modeling technique
Factor Type(s)	age • sex • education level • Shared Socioeconomic Pathway • year • province
Sample Characteristic - Location	China

Machine-accessible metadata file describing the reported data: 10.6084/m9.figshare.11898405

## Background & Summary

Population has direct influences on the challenges related to the mitigation of and adaptation to climate change by influencing economic growth and social development, affecting the amounts of resource consumption and pollutant emissions, and determining the number of residents exposed to pollutants and natural disasters^1–5^. As China is the most populous country in the world, estimations of China’s population and its spatial distribution is important in global and China’s scenario researches.

Despite the significance and demand for refined population studies, research on China’s future population, especially the provincial population, is not sufficient. The International Institute for Applied Systems Analysis (IIASA) created a projection of population in China at the national scale from 2010 to 2100 under the shared socioeconomic pathways (SSPs) in 2017^6,7^. This study assumed a total fertility rate (TFR) of 1.4 from 2010 to 2070 and a rate of 1.5 afterwards under medium assumption of fertility, and even under the assumption of high fertility, it does not exceed 1.8. However, due to the implementation of the transitional selective two-child policy in China in November 2013 and the two-child policy in January 2016, i.e., all families are allowed to have two children without any restriction, the downward trend of TFR has been slightly reversed from 1.18 in 2010 to 1.7 in 2016^8^, which could be viewed as a new business-as-usual TFR in China. Besides, the IIASA projection also assumed that the life expectancy at birth (LE) of women would reach 93.2 (84.7, 101.7) in 2100 under the medium (low, high) scenario for China, which is much higher than medium (low, high) estimation made by the UN Population Division of 88.2 (83.4, 92.4). These systematic biases in the fertility rate and life expectancy assumptions made it necessary to update the projections of China’s population to be better in line with the actual situation.

To meet the further demands of health risk, employment impact, and inequality research, there is also an emerging need for sub-national population projections with detailed attributes of age, sex and educational attainment as well as for the corresponding gridded datasets. Currently, refined sub-national population prediction studies have been developed in the United States^9^, and grid-level population for Africa and the world have been established based on existing national population projections such as SSPs and the IPCC Special Report on Emission Scenarios (SRES)^10–12^. However, for China, the population projections available from the IIASA SSP database represent the national-level population and the national no-education population for 21 five-year age groups by sex every five years from 2010 to 2100^7^, and dataset is lacking at the provincial and the grid levels. Although a domestic Chinese institution has provided a provincial-scale estimate^13^, to the best of our knowledge, there is no publicly available provincial or grid-scale estimation in China that considers the latest changes of the fertility rate and can also reveal details in sex, age and educational attainment. This limitation has caused constraints for some research^14^. In addition, another policy in China – the population ceiling policy - will have a substantial impact on the distribution of the population, but the existing research has not considered this policy. Population ceiling policies, i.e., restrictions of the population size within a city, have been introduced in several megacities. For example, the number of permanent residents in Beijing will be controlled to less than 23 million by 2020 by adjusting the settlement policy^15^, while the population in Shanghai will be mandated to be controlled within 25 million by 2035^16^.

Therefore, our research aims to compensate for this problem by estimating the yearly provincial population in China with specific demographic information on sex, age and seven educational attainment categories (consistent with the categories in the Chinese census) under SSPs from 2010 to 2100, while considering the changes in national fertility policies and population ceiling policies. In addition, we downscaled the projected provincial population and established spatially explicit population grids for China with a resolution of 1 km from 2010 to 2100 under SSPs to meet the requirements of integrated assessment models. Both the provincial and gridded data will be useful for studies and simulations in future climate policy, public health, resource demand and allocations, environmental impacts and social equity.

## Methods

**Fig. 1:**
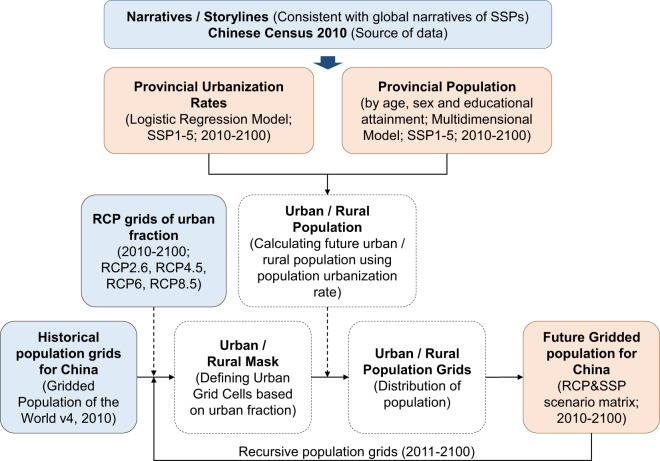
Methodology Framework for Provincial Population Projection and Downscaling. This study, which was based on a global narrative of SSPs and data from the Chinese Census 2010, used a recursive multidimensional model to project provincial populations in China under five SSPs and distributed them into spatial grids. The blue rectangles contain exogenous data, the red rectangles are the research outcomes from this study, and the white ones describe the modelling steps.

In accordance with the main steps in developing SSPs including developing narratives, quantifying scenario assumptions, and elaborating socioeconomic scenario drivers (i.e., population, GDP, urbanization) via quantitative models^6^, the methodology framework is shown in Fig. 1. This study first describes the demographic assumptions at the provincial level based on SSP storylines, and then demonstrates the methods in provincial population projection, urbanization estimation and population downscaling.

### Demographic assumptions under SSPs

SSP1 depicts a sustainable development scenario in which investment in education and health enhances demographic transition in all provinces, resulting in a relatively low population in China. Thus, fertility is assumed to be low and current fertility policies are not effective enough and only temporarily relieve the decline in the TFR by 2020. Mortality will decline, and educational attainment will rapidly increase. Because of the reduced inequality within China, provincial migrations are assumed to be moderate in all provinces.

SSP2 can be considered a business-as-usual scenario that maintains historical development features. As a result, the fertility rate is assumed to be moderate in the future under an effective two-child policy. Mortality and provincial migration are both at the medium level. Educational attainment in each province will maintain the same historical growth rate.

SSP3 is a global regional rivalry scenario. The competitiveness among countries will result in security-focused national development policies. Population growth in China will be high with an effective, fully open fertility policy to ensure the better reservation of human capital. Low investments in education and health will result in high mortality and low educational attainment. Although inequality will worsen, low economic growth in all provinces will result in decreasing migration between provinces.

SSP4 describes an inequality scenario in which a polarized educational attainment pattern will appear among and within countries, with increased educational growth in well-developed provinces and a constant progression rate in provinces with poor economic development. Provinces will show different demographic patterns, and the country as a whole will experience low fertility and mortality rates, consistent with the global assumptions. The inter-provincial migration will be considered moderate, because it will be affected by the opposite directions of severe regional inequality and low economic growth among provinces.

In SSP5, which is the fossil-fueled development scenario, the country will pay attention to educational investment, resulting in low fertility and mortality rates. The active technology and capital market will encourage migration between provinces. Table 1 summarizes the assumptions of fertility, mortality, migration, educational attainment, and population policies, which are the key factors impacting the future population.

**Table 1:** Demographic assumptions in China under the five SSPs.

Scenarios	Fertility	Mortality	Migration	Education	Policies	
SSP1	Low	Low	Medium	High	Ineffective fertility policy	Population ceiling policy in megacities
SSP2	Medium	Medium	Medium	Medium	Effective two-child policy	
SSP3	High	High	Low	Low	Effective fully open policy	
SSP4	Low	Medium	Medium	H/M/L*	Ineffective fertility policy	
SSP5	Low	Low	High	High	Ineffective fertility policy	

*In SSP4, assumptions for educational attainment depend on the provincial development level. Details can be found in the provincial population projection section.

### The projection of provincial population

A recursive multidimensional model is used to project the provincial population with details on age, sex and educational attainment. Equations 1a and 1b describe the newborn population of a certain province, which is determined by the corresponding population by age and education in the previous year, the educational fertility rates of women at childbearing age, and the neonatal sex ratio.

1a $$P{m}_{yr,a=0,edu=1}=\mathop{\sum }\limits_{a=15}^{49}(\mathop{\sum }\limits_{edu=1}^{7}(P{f}_{yr-1,a,edu}\times FE{R}_{yr,a,edu}))\times B{m}_{yr}$$

1b $$P{f}_{yr,a=0,edu=1}=\mathop{\sum }\limits_{a=15}^{49}(\mathop{\sum }\limits_{edu=1}^{7}(P{f}_{yr-1,a,edu}\times FE{R}_{yr,a,edu}))\times B{f}_{yr}$$

*Pm* and *Pf* represent the male and female populations respectively, *FER* is the fertility rate, and *Bm* is the ratio of newborn males (*Bf* for females). The subscript *yr* is a certain year from 2011 to 2100, *a* is a certain age from 0 to 99 and a category named “100 and above” (shorted as “100+”), and *edu* symbolizes the seven stages (from 1 to 7) of education in China, which are consistent with results in the census: illiterate, primary education (aged 6–12), junior high school (aged 13–15), senior high school (aged 16–18), college education (aged 19–20), bachelor’s education (aged 19–22), master’s education and above.

Equations 2a and 2b are the most common situations for iterating the provincial population from one-year-old to “100+” with different educational attainments.

2a $$\begin{array}{lll}P{m}_{yr,a+1,edu} & = & \left(P{m}_{yr-1,a,edu}\times (1-MOR{m}_{yr,a,edu})\times (1+NetPI{M}_{yr,a,edu})\right.\\  &  & \times \,(1-G{m}_{yr,a,edu})+P{m}_{yr-1,a,edu-1}\times (1-MOR{m}_{yr,a,edu-1})\\  &  & \left.\times \,(1+NetPI{M}_{yr,a,edu-1})\times G{m}_{yr,a,edu-1}\right)\times (1+NetGI{M}_{yr})\end{array}$$

2b $$\begin{array}{lll}P{f}_{yr,a+1,edu} & = & \left(P{f}_{yr-1,a,edu}\times (1-MOR{f}_{yr,a,edu})\times (1+NetI{M}_{yr,a,edu})\times (1-G{f}_{yr,a,edu})\right.\\  &  & {\rm{+}}\,P{f}_{yr-1,a,edu-1}\times (1-MOR{f}_{yr,a,edu-1})\times (1+NetPI{M}_{yr,a,edu-1})\\  &  & \left.{\rm{\times }}\,G{f}_{yr,a,edu-1}\right)\times (1+NetGI{M}_{yr})\end{array}$$

$MORm$ is the mortality rate for males (*MORf* for females), $NetPIM$ is the net provincial immigration rate, $Gm$ is the progression rate between different educational levels for males (*Gf* for females), and $NetGIM$ is the net global immigration rate of China. The population of a certain education stage is composed of people who graduate from an earlier education stage and those who do not graduate and remain in the same stage.

A special situation exists in both the illiterate population (*edu* = 1), which does not have an earlier education stage, and the population aged “100+” (*a* + 1 = 100), which considers the population aged both 99 and “100+” in the previous year. Details on the special situations are provided in the Supplementary Information. Data used in this recursive multidimensional model come from Chinese census 2010.

In the medium-fertility assumptions, the initial TFR in 2010 is adjusted to 1.6 according to the results of studies that found that China’s TFR in the 2010 census was seriously underestimated^17–21^. Due to the newly released fully open two-child fertility policy, the TFR in China in medium fertility scenario is estimated to peak at 1.8 in 2020, restore to a normal policy incentive level of 1.65 in 2030 after fully releasing the cumulative effect of the fertility policy, and maintain the same growth rate as the UN’s medium estimation of TFR from 2030 (1.706) to 2100 (1.802)^22^. The high fertility scenario assumes that the country will carry out more radical fertility policies, i.e., the fully open policy, resulting in the TFR reaching 2 by 2020, being 25% higher than the medium in 2050, and remaining constant afterwards. The low fertility scenario assumes that the current fertility policy can only suspend the decreasing trend of TFR in recent years, and is not efficient enough to change the decreasing trend of the TFR in the long term. As a result, under the low fertility assumptions, the TFR increases from 2016 to 2020, decreases by 25% in 2050 compared to the medium, and remains the same after 2050. The matrix of the fertility rate for the different education levels by age (15–49, the childbearing age of women) and province are separately set based on the same proportions in the 2010 census, as shown in Suppl. Table 1. In addition, the sex ratios at birth by province are assumed to reach 1.07 in 2050, and remain constant after 2050, according to National Population Development Plan with a long-term target of 1.07^23^.

The medium mortality assumes that the LE in each province will increase at the base rate of one year per decade, adjusting by the initial ratio of the provincial LE to the national LE in 2010. Under this scenario, the LE in China will experience an average growth of 0.96 years per decade and reaches 87, the average UN’s medium LE estimation of different gender, in 2100^22^. Referring to KC and Lutz, and Jiang *et al*.’s approach to setting the high and low mortality scenarios^7,13^ and making the assumptions in a relative reasonable range, the provincial LE in the high mortality scenarios is set to grow at a basic rate of 0.5 years per decade and will be adjusted by the initial ratio, while the basic growth rate in the low scenario is 1.5 year per decade. The national LE in 2100 under the high and low mortality assumptions are 82 and 91 respectively, matching the average UN’s estimation^22^. The 2010 census serves as the data source for calculating the base-year LEs and setting the matrix of mortality rates by age, sex and province (Suppl. Table 2).

In terms of provincial migration, provinces are divided into three different categories because provinces at different economic development stages have different trends of migration changes. The classification method refers to the article of Ding and Zhong^24^. Eight provinces with high income have implemented or will implement population ceiling policies to control excessive population concentration and promote regional equality. Low-income provinces have relatively high negative net migrations because economic status and settlement policies are more attractive in other provinces. The migration rate in medium-income provinces will increase gradually with more policies that attract talent in these provinces.

The assumptions under the three scenarios in each category are described in Table 2. Recognizing that the assumptions of provincial migration rates, rather than assumptions of absolute migration flows, can produce unbalanced migration population, i.e. the inter provincial migration population does not equal zero, the unbalanced excess migration is cut back at the same proportion of provincial migration in the calculation process.

**Table 2:** Assumptions of migration scenarios in each income category.

Income categories	Assumptions of migration scenarios			Province list
	High	Medium	Low	
High	Zero $NetPIM$ in 2010 until 2100	Zero $NetPIM$ in 2020 until 2100	Zero $NetPIM$ in 2030 until 2100	Beijing, Tianjin, Inner Mongolia, Shanghai, Jiangsu, Zhejiang, Fujian, Guangdong
Medium	50% of current $NetPIM$ in 2100	50% of current $NetPIM$ in 2050, 0 in 2100	50% of current $NetPIM$ in 2030, 0 in 2100	Hebei, Jilin, Heilongjiang, Anhui, Jiangxi, Shandong, Henan, Hubei, Hunan, Hainan, Chongqing, Sichuan, Shaanxi, Qinghai, Ningxia, Xinjiang
Low	150% of current $NetPIM$ in 2050 and remain constant	Constant $NetPIM$	50% of current $NetPIM$ in 2050 and remain constant	Shanxi, Liaoning, Guangxi, Guizhou, Yunnan, Tibet, Gansu

Although international migration rates differ between provinces in reality, this study assumes that all provinces have the same international net immigration rate as the country due to data availability. The net immigration rate in 2010 is −0.3015‰, which is the average of −0.3570‰ (2005–2010) and −0.2460‰ (2010–2015) in the UN’s estimates of migration^22^. This rate remains constant in the second half of the century and gradually decreases to zero in 2100 under the medium assumption. The high and low scenarios are 50% larger and smaller than the medium scenario, respectively.

In the medium educational attainment scenario, the progression rates (PRs) among seven educational levels by gender remain at the historical growth rate in each province. The low scenario maintains the same PR in 2010, and the high scenario applies the currently largest growth rate in all provinces. The PRs by education, sex and province in 2010 are based on the census data (Suppl. Table 3). A maximum constraint is set for each rate. Enrolment rates for primary school, the PRs from primary school to junior high, and those from junior to senior high school are not greater than 99.9%, because of the 9-year compulsory education policy has taken effects in all provinces and a 12-year compulsory education policy is under discussion. The maximum PRs are 30% for senior high school to college education, 60% for senior high school to undergraduate studies, and 30% for graduate studies, according to the average calculated PRs after senior high school in Korea, whose basic education is well developed, from 2013 to 2016 which are 29% and 58% respectively^25^, as well as the master entry rate reaching 29% in 2017 in the U.K. with better second stage of tertiary education. The PRs remain unchanged after reaching the maximum value. In SSP4, the provinces with different income levels are assumed to attain different educational development because of the high inequality within the country, i.e., medium/low/high-income provinces experience medium/low/high education scenarios. The three categories of provinces are the same as those in Table 2.

### Calculating the provincial urban and rural population using the projected urbanization rate

Various indicators can be used to measure the rate of urbanization, including economic index, urban population ratio, and the total built-up area^26^. Here, we constructed datasets of future provincial population urbanization rates for China under five SSPs from 2010 to 2100 and used them to calculate the yearly provincial urban and rural population.

The urbanization projections employed an annual database of provincial urbanization levels that extended from 1995–2015 for 31 provinces in China. The measures of urbanization rate are defined as the percentage of the urban population by total provincial population. The data from 1995 to 1999 is from Zhou and Tian^27^ and 2000–2004 from Lin^28^, both of whom mended the data of census and provided a consistent measurement of urbanization levels for each province. The remaining data (2005–2015) originate from China Statistical Yearbook series^29^. As shown in Eq. 3, the urbanization level for each province is projected as a sigmoid function of time, depicting features of an S-shape curve – the urbanization rate initially grows faster, and the growth rate slows at the middle stage until it finally stops growing^30^.

3 $$PU=\frac{b}{1+ex{p}^{-c(T-d)}}$$

Here, *PU* is the population urbanization rate, which is defined as the proportion of the total urban population with respect to the total population, *T* stands for the time span of the projection (the projected year minus the base year), and *b* is a parameter of the upper limit of the urbanization rate. The coefficient *c* denotes the specific rate or pace at which urbanization level changes with time, while *d* represents the time of the inflection point when the *PU* reached the half of the logic curve. The annual increments in urbanization levels become increasingly larger between *T* = 0 and *T* = *d*, then they become increasingly smaller until *T* = *T*_*max*_^31^. These two parameters are estimated by ordinary least squares (OLS) regression.

To remain consistent with urbanization assumptions of each SSP narrative^32^, we built sigmoid functions for each province under fast, medium and slow developing assumptions. First, the upper limits (parameter *b* in the function) of the urbanization rates for each projected province were set based on their urbanization rate in 2015, as shown in Table 3. For example, if the urbanization rate of a province is between 60% and 70% in 2015, the upper limits under the medium assumptions would be 80%.

**Table 3:** The upper limits (b) of urbanization rates under each assumption.

Criterions	Fast assumptions	Medium assumptions	Slow assumptions
*PU*_2015 _ ≥ 70%	100%	100%	100%
60% ≤ *PU*_2015_ < 70%	90%	80%	75%
*PU*_2015_ < 60%	85%	80%	70%

*PU*_2015_ denoted the urbanization rate of the projected province in 2015.

Then, in order to select a set of referencing provinces to develop the sigmoid function to project the urbanization level under the fast (slow) assumptions for each projected province, we take two steps (Table 4). First, we choose the provinces whose urbanization rates in 2015 were 5% higher (lower) than the target provinces as the initial referencing sets. Second, we eliminated the provinces under slower (faster) urbanization processes by excluding those with smaller (larger) urbanization rate changes between 1995 and 2015 than those in the projected province. This step ensures the similarity in terms of urbanization growth rates between reference provinces and the projected province.

**Table 4:** The selection process for referencing provinces for urbanization projections under fast and slow assumptions.

Scenarios	Included	Excluded
Fast assumptions	*PU*_2015_ < *R*_2015_ < *PU*_2015_ + 5%	Δ*R* < Δ*PU*
Slow assumptions	*PU*_2015_ − 5% < *R*_2015_ < *PU*_2015_	Δ*R* > Δ*PU*

*PU*_2015_ is the urbanization rate of the province to be projected in 2015, while *R*_2015_ denotes the urbanization rate of the referencing province. Δ indicates the increase in urbanization during 1995–2015, e.g. Δ*R* denotes changes in urbanization of reference provinces between 1995 and 2015.

For the medium assumption, we did not set referencing provinces for the projected province and assumed that its urbanization growth followed its own historical trend. Specifically, we defined the sigmoid function for each province based on its historical urbanization rates during 1995–2015 and projected its future urbanization level.

Finally, the provincial urban population by years for China, as well as the rural population (subtracting urban population from total population), is calculated by multiplying the provincial projection results of the population and population urbanization rates, which become the basic input data for developing the population grids.

### Downscaling the provincial population

Downscaling the population from the national or provincial level into high-resolution grids, while distinguishing between urban and rural populations, usually follows three steps. First, identify the urban grid cells in each administrative area based on the last-year population grid map with certain rules; second, calculate the total urban and rural populations to be allocated, according to the projection results for the population and population urbanization rates; third, establish methods to determine the distributional weight such as the constant share of the population in each grid cell^10,33,34^, share of population growth in each grid cell^35^, and share of the gravity changes in the gridded population^11,36^.

**Fig. 2:**
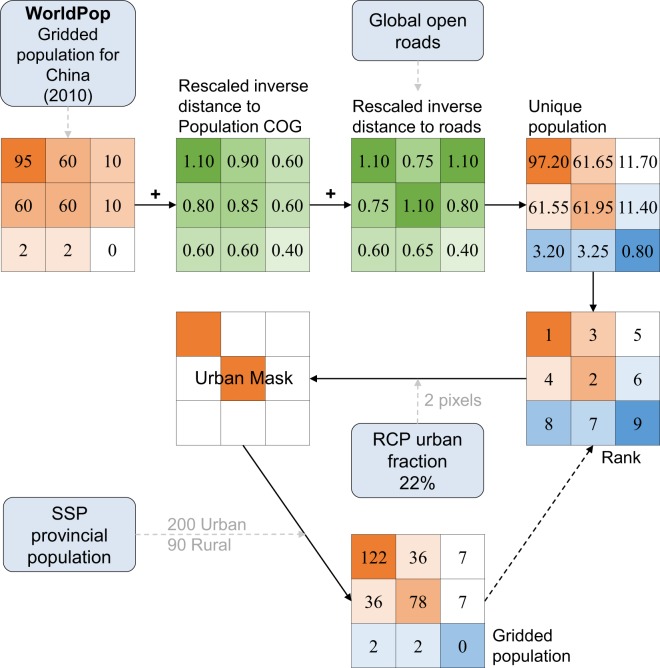
Schematic diagram of downscaling methods to produce population grids. Conceptual overview of the downscaling approaches including key steps in generating the basic unique population grids for 2010, and mapping population based on RCP urban fraction and SSP provincial population (modified from Niklas Boke-Olén *et al*.^12^).

This study uses the methods in Boke-Olén *et al*.’s research, as shown in Fig. 2, which downscales the national population in Africa under SSPs into population grids at 1 km resolution from 2010 to 2100^12^. The basic gridded population for China in 2010 was generated from the WorldPop Gridded Population^37^ at 30″ × 30″ (approximately 1 km resolution at the equator) by removing waterbodies, and adding the rescaled inverse distance to roads and the population centre of gravity lying between 1.0 × 10^−5^ and 1.1 × 10^−5^ ^12^. Here, the inverse distance to roads is the Euclidian distance on the Global Roads Open Access Data Set^38^, while the population centre of gravity is calculated using COGravity tool in R on WorldPop datasets. We use the RCP urban fraction data^39^, which provide the proportion of urban land within each 0.5° (approximately 50 km at the equator) grid cell, to classify each 30″ grid cell within each 0.5° grid cell as urban or rural. The pixel values in the basic population grid map in 2010 are sorted, and the highest value is selected until the number of grid cells defined as urban areas corresponds to the nearest integer of the calculated urban pixel numbers within the cell, i.e. the number of pixel selected as urban grid cells corresponds to the urban fraction in each 0.5° grid cell. The provincial urban population is allocated within the urban mask, while the rural population is distributed throughout non-urban grids. Here we select 15 of 20 scenario matrices constitute by the four RCP scenarios (RCP2.6, RCP4.5, RCP6 and RCP8.5) and five SSP scenarios that are more likely to happen from K. Engström *et al*. (Table 5), i.e. the quantitative probabilities are larger than zero^40^.

**Table 5:** Quantitative probabilities of RCP-SSP scenario matrix from K. Engström *et al*.^40^.

	RCP2.6	RCP4.5	RCP6	RCP8.5
SSP1	0.0909	0.4545	0.4545	0.0000
SSP2	0.0000	0.0909	0.6818	0.2273
SSP3	0.0000	0.1667	0.5000	0.3333
SSP4	0.0000	0.3704	0.5556	0.0741
SSP5	0.0000	0.0741	0.3704	0.5556

A recursive process is developed to build the next population grids, i.e., the gridded population from the previous year is used to generate the urban mask for the following year.

## Data Records

The projected yearly provincial population by age, sex, and education attainment, under SSPs for China from 2010 to 2100, the projected yearly provincial population urbanization rates under SSPs (2010–2100), and the future spatially explicit population maps are all available at the public repository Figshare^41^.

Provincial population data with specific demographic attributes for a certain year and SSP are stored in the file “Pop_E_provincename_SSPx_year.csv”, while x represents each scenario number. The file contains two sex groups (M = Male, F = Female), seven educational levels (E0 = Illiterate, E1 = Primary school, E2 = Junior high school, E3 = Senior high school, E4 = College education, E5 = Bachelor’s degree, E6 = Master’s degree and above), and 101 different age groups (0, 1, 2… 99, 100+). In addition, the total population of each province, the year and the scenario, which are most commonly used in further climate policy studies, are summarized in the file “Pop_TOTAL.csv”.

Future spatially explicit population grids under the RCP-SSP matrices for each scenario and year are stored as a GeoTIFF file (.tif), with the WGS84 projection at 30 arc-seconds resolution.

## Technical Validation

The projected provincial population takes 2010 as the base year. Among all five SSP scenarios, SSP2 (named “Middle of the Road”) usually follows the historical development trends and is considered the business-as-usual (BAU) scenario in climate policy research. As a result, the technical validation mainly focuses on the results of the projection under the SSP2 scenario.

Here, we use absolute percentage error (APE) and algebraic percentage error (PE), which are commonly used accuracy indicators in population projections^9,42,43^, to evaluate the predictive accuracy and bias in the provincial total population, age structure and educational attainment. The equations for the indicators are as follows, where *P*_*t*_ is the projected result and *A*_*t*_ represents the actual value.

4 $$APE) \% )=\left|\frac{{P}_{t}-{A}_{t}}{{A}_{t}}\right|{\rm{\times }}\,100 \% $$

5 $$PE( \% )=\left(\frac{{P}_{t}-{A}_{t}}{{A}_{t}}\right){\rm{\times }}100 \% $$

Therefore, projection is overestimated when PE is positive, and vice versa. All actual values that are used for validation are from the latest China Population and Employment Statistical Yearbooks based on the 1% sample census in 2015 and 1‰ sample census in 2016 and 2017^44^. Then, we evaluate the accuracy of the population grids by aggregating the grids into prefecture-level cities and counties in China and comparing the results with actual statistical population data from provincial and prefecture-level Economic Statistics Yearbook in 2015. We also compare the generated population grids with existing population grids products.

### Errors in provincial population projection

Table 6 shows the predictive errors in the national and provincial population projections based on the total population in the statistical yearbooks from 2015 to 2017. For the national population estimation, our projection is 0.7% − 0.9% larger than the actual value. For the provincial projection, the mean APE of all 31 provinces ranges from 1.7% to 2.0%, and the projection is slightly overestimated with a positive mean PE.

**Table 6:** Errors in the total population projection.

Region	Indicator	2015	2016	2017
National	APE (%)	0.7	0.8	0.9
	PE (%)	0.7	0.8	0.9
Provincial	Mean APE (%)	1.7	1.9	2.0
	Mean PE (%)	0.5	0.6	0.7

As shown in Table 7, most provinces have relatively low APEs, and all provinces have APEs below 5% in 2017. Xizang, Chongqing and Tianjin are underestimated with errors over 4%, while the projected populations in Hunan, Qinghai, Shandong, etc. are more accurate with APEs less than 1%.

**Table 7:** APEs of the provincial population projection in 2017.

Provinces	APE(%)	Provinces	APE(%)	Provinces	APE(%)
Beijing	1.9	Anhui	1.3	Chongqing	4.5
Tianjin	4.2	Fujian	1.0	Sichuan	2.4
Hebei	1.1	Jiangxi	1.0	Guizhou	1.9
Shanxi	0.8	Shandong	0.5	Yunnan	1.1
Inner Mongolia	2.3	Henan	4.0	Tibet	4.8
Liaoning	2.3	Hubei	1.5	Shaanxi	1.3
Jilin	2.4	Hunan	0.1	Gansu	1.9
Heilongjiang	2.1	Guangdong	2.6	Qinghai	0.1
Shanghai	2.8	Guangxi	1.3	Ningxia	0.8
Jiangsu	2.7	Hainan	4.0	Xinjiang	0.8
Zhejiang	3.0				

Table 8 shows the APEs and PEs of the different age groups. The 1‰ sample census does not reveal information on provincial 1-year age groups, while nearly half of the provinces have not published results from the provincial 1% sample census. Due to data availability, we use the proportion of the population in each five-year age group in the national sample census to verify the age information of our projected population. There are larger deviations in the population proportion of the low-age and high-age groups, while those in the middle-age groups are underestimated. The results of the errors are acceptable compared with the errors in the age structure of a population projection for the U.S in 2015^9^.

**Table 8:** Errors in national age structure.

Age Group	APE (%)			PE (%)		
	2015	2016	2017	2015	2016	2017
0–4	8.2	7.1	6.3	8.2	7.1	6.3
5–9	1.1	0.7	2.3	−1.1	0.7	2.3
10–14	1.0	1.1	1.2	−1.0	−1.1	−1.2
15–19	0.3	0.3	0.2	0.3	0.3	0.2
20–24	1.2	1.2	1.2	−1.2	−1.2	−1.2
25–29	1.7	1.9	1.9	−1.7	−1.9	−1.9
30–34	1.3	1.5	1.7	−1.3	−1.5	−1.7
35–39	1.1	1.2	1.4	−1.1	−1.2	−1.4
40–44	0.9	1.0	1.1	−0.9	−1.0	−1.1
45–49	0.8	0.9	1.0	−0.8	−0.9	−1.0
50–54	0.7	0.8	0.9	−0.7	−0.8	−0.9
55–59	0.6	0.6	0.6	−0.6	−0.6	−0.6
60–64	0.3	0.5	0.7	−0.3	−0.5	−0.7
65–69	0.1	0.1	0.3	0.1	0.1	−0.3
70–74	1.3	1.1	2.1	1.3	1.1	2.1
75–79	3.0	4.3	3.8	3.0	4.3	3.8
80+	10.5	10.8	11.1	10.5	10.8	11.1

This table shows APEs and PEs in the different population proportion for 17 five-year age groups (0–4, 5–9… 75–79, 80+).

The errors in the projection of educational attainment are shown in Table 9. Here, we calculate the APEs and PEs based on the population proportion of different educational levels provided in the sample census. The APEs of national educational attainments are relatively small within the range of 0% to 3.3% (0% to 4.2% for the provincial mean). The population proportions with higher education levels (college+) are underestimated with relatively larger errors.

**Table 9:** Errors in the projection of educational attainment.

Year	Region	Indicator	Illiterate	Primary	Junior	Senior	College+
2015	National	APE (%)	0.9	0.2	0.0	1.5	2.3
		PE (%)	−0.9	0.2	0.0	1.5	−2.3
	Provincial	Mean APE (%)	1.0	0.3	0.1	1.9	2.5
		Mean PE (%)	−0.9	0.2	0.0	1.8	2.5
2016	National	APE (%)	0.5	1.0	0.3	1.7	3.3
		PE (%)	0.5	1.0	0.3	1.7	−3.3
	Provincial	Mean APE (%)	0.5	0.5	0.4	1.5	4.2
		Mean PE (%)	−0.1	0.3	−0.4	1.3	−4.2
2017	National	APE (%)	0.1	1.4	1.0	3.1	2.2
		PE (%)	−0.1	1.4	1.0	3.1	−2.2
	Provincial	Mean APE (%)	1.2	0.4	0.1	2.4	3.4
		Mean PE (%)	−1.2	0.4	0.0	2.3	−3.4

Here we represent the errors in five categories of educational attainment: illiterate, primary school, junior high school, senior high school, and college and above.

**Fig. 3:**
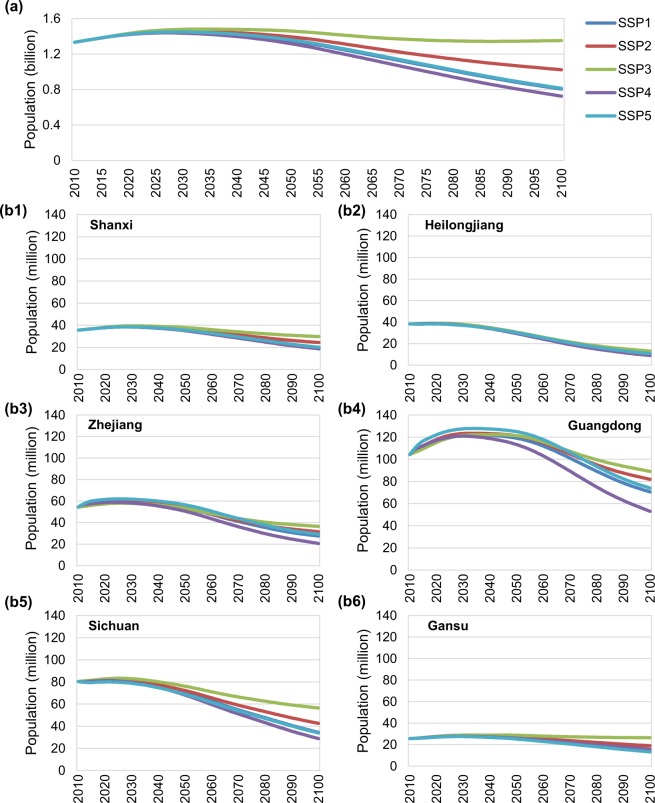
Changes in national and provincial populations under SSPs. This figure demonstrates (**a**) future population changes at the national level from 2010 to 2100 under five SSPs, and (**b1**–**b6**) the population changes in sample provinces (i.e., Shanxi in North China, Heilongjiang in Northeast China, Zhejiang in East China, Guangdong in South Central China, Sichuan in Southwest China, and Gansu in Northwest China) between 2010 and 2100 under five SSPs.

Figure 3 shows the future trends of the national population and sample provincial population from 2010 to 2100 under SSPs. China usually divides provinces into six regions according to geographical distribution, namely North China, Northeast China, East China, South Central, Southwest China, and Northwest China. Here, we select six provinces including Shanxi, Heilongjiang, Zhejiang, Guangdong, Sichuan and Gansu as the sample provinces in each region. The national population will reach a peak of 1.46 billion (with a range of 1.44 billion to 1.48 billion) in 2029 (2027–2034). Except for SSP3, which showed a continuous increasing trend, the national population showed a significant downward trend in the other four scenarios. By 2100, the national population was estimated to be 0.72 to 1.35 billion. Different provinces have different demographic trends due to differences in their base-year situations, developing capacities and implemented policies.

### Validation of population grid maps

First, we check the consistency of the distribution by comparing the aggregated population grids in each province with the projected provincial population. The results are the same in each year under each scenario, which shows that the gridded population allocated by this method is consistent with the projected value.

Then, the reliability of the gridded population is analysed by comparing the generated 2015 population grids under SSP2, which is considered a business-as-usual scenario, with finer-level statistical resident population data, i.e. prefecture-level and county-level population data, from provincial and prefecture-level Economic Statistical Yearbook. The population of gridded maps of SSP2 RCP4.5, SSP2 RCP6 and SSP2 RCP8.5 are aggregated into 339 prefecture-level cities and 2858 counties in China according to administrative divisions in 2015 generated by National Geomatics Center of China (NGCC)^45^. Due to the availability of resident population at finer-levels, we compare the data in 317 prefecture-level cities and 2579 counties. Root mean square error (RMSE), %RMSE (RMSE expressed as a percentage of the average population at finer-levels), mean absolute error (MAE) and median absolute deviation (MAD) are used to describe the performance of grid maps, referring to statistical criteria used in technical validation of gridded population products by Gaughan, A.E. *et al*.^46^ and Sorichetta, A. *et al*.^47^. Table 10 summarizes the results of the four criteria calculated at both population unit counts (the number of people in an administrative unit) and population densities (population unit counts divided by the number of pixels from the population map within the corresponding administrative unit). The volume of RMSE, MAE and MAD are affected by administrative level, for the average population size at different administrative tiers varies, while %RMSE is a dimensionless indicator that is more reasonable to be used for comparisons between validations. The %RMSE in validations at prefecture-level and county-level for population unit counts are 17.6%-23.5% and 39%-49.1% respectively, which is acceptable comparing with the %RMSE in studies^46,47^.

**Table 10:** Results of statistical criteria in evaluating the accuracy of the generated population grids under SSP2 in 2015 at prefecture-level and county-level.

Aggregated administrative levels	Scenarios	Criteria			
		RMSE	%RMSE	MAE	MAD
***Population unit counts***					
Prefecture-level Cities	SSP2RCP4.5	974479.9	23.5	588863.2	326479.7
	SSP2RCP6	731141.4	17.6	448259.4	255456.4
	SSP2RCP8.5	805875.8	19.4	490549.0	273707.3
Counties	SSP2RCP4.5	247844.4	49.1	145966.7	82467.7
	SSP2RCP6	196881.3	39	114538.7	63821.5
	SSP2RCP8.5	213098.9	42.2	125072.7	70489.5
***Population densities***					
Prefecture-level Cities	SSP2RCP4.5	156.5	49.2	53.1	18.3
	SSP2RCP6	111.3	35.0	39.5	15.0
	SSP2RCP8.5	125.5	39.5	43.5	15.5
Counties	SSP2RCP4.5	2112.8	240.7	496.3	37.4
	SSP2RCP6	1669.5	190.2	391.1	29
	SSP2RCP8.5	1810.8	206.3	424.1	30.8

We also make a cell by cell comparison between the generated population grids of SSP2RCP4.5, SSP2RCP6 and SSP2RCP8.5 in 2015 with the world population grids in 2015 produced by WorldPop^37^, and the Gridded Population of the World (GPW) v4^48^. In general, differences are relatively small in most areas and they are larger in densely populated urban areas. We summarize the results of several criteria including RMSE, MAE, MAD, mean APE and median APE in Table 11 to evaluate the differences.

**Table 11:** Results of statistical criteria in evaluating the differences between population grids products in 2015.

Scenarios	Criteria				
	RMSE	MAE	MAD	Mean APE(%)	Median APE(%)
***Comparing with WorldPop***					
SSP2RCP4.5	603.2	58.2	1.4	45.4	43.2
SSP2RCP6	469.6	49.7	1.3	43.5	40.4
SSP2RCP8.5	518.7	53.0	1.4	44.3	41.6
***Comparing with GPWv4***					
SSP2RCP4.5	746.4	83.4	3.8	79.9	67.9
SSP2RCP6	627.1	78.0	3.7	80.3	66.7
SSP2RCP8.5	672.3	80.2	3.8	80.3	67.2

## Usage Notes

SSP scenarios are designed to depict different future development directions and reduce uncertainties by providing a reference range for the major socioeconomic drivers. During the prediction process, we comprehensively consider the impacts of many factors, such as birth, death, migration and education level, and provide a range of future changes in provincial populations with detailed attributes for sex, age and educational attainment under five SSPs. However, there are still uncertainties, especially policy uncertainties, which will influence the results of both the projection and distribution. For instance, China has implemented population ceiling policies in megacities to reduce provincial immigration by establishing more strict settlement regulations. Our model simulates the possible impacts of these current policies, but it does not take into account the possible adjustments of these policies after populations in megacities reach their peaks and start to decline.

In addition, with the increase in the urbanization rate in all provinces, the urban population growth is obvious. The Chinese government at all levels has implemented and may plan to introduce new town or new district construction policies to reduce and relieve “urban disease” such as severe traffic congestion, natural resources shortages and pollution, which are brought about by the increased urban population density. For example, China has proposed the establishment of a new district in Hebei Province and moving the administration to an east suburb district of Beijing to ease population pressure in the central region of the capital. The implementation of these policies will lead to the redistribution of population, but the current gridding method based on the historical population distribution does not consider these impacts.

## Supplementary information

Supplementary information

Suppl. Table 1

Suppl. Table 2

Suppl. Table 3

## Data Availability

All R codes (R3.5.3, https://www.r-project.org) for creating provincial and gridded population datasets for China are stored in public repository Figshare^41^. Explanations are internalized in the script to help users with implementation.
