# Supplementary material for: Provincial and gridded population projection for China under shared socioeconomic pathways from 2010 to 2100
Source: Sci Data. 2020 Mar 9;7:83. doi: 10.1038/s41597-020-0421-y (PMC7062824; doi:10.1038/s41597-020-0421-y)
Supplement: Supplementary file 1 — Supplementary information [file 41597_2020_421_MOESM1_ESM.docx]

**Provincial and gridded population projection for China under shared socioeconomic pathways from 2010 to 2100**

Yidan Chen^1^, Fang Guo^1^, Jiachen Wang^1^ , Wenjia Cai^2,3,4^, Can Wang^1,4^ & Kaicun Wang^5^

**Affiliations**

1. State Key Joint Laboratory of Environment Simulation and Pollution Control (SKLESPC), School of Environment, Tsinghua University, Beijing 100084, China

2. Ministry of Education Key Laboratory for Earth System Modeling, and Department of Earth System Science, Tsinghua University, Beijing, 100084, China

3. Center for Healthy Cities, Institute for China Sustainable Urbanization, Tsinghua University, Beijing 100084, China

4. Tsinghua-Rio Tinto Joint Research Center for Resource Energy and Sustainable Development, Tsinghua University, Beijing 100084, China

5. College of Global Change and Earth System Science, Beijing Normal University, 19 Xinjiekouwai Street, Haidian, Beijing, 100875, P. R. China

corresponding author(s): Wenjia Cai (wcai@tsinghua.edu.cn)

**Supplementary Information**

# Special situations for iterating the provincial population

A recursive multidimensional model is used to project the provincial population with details on age, sex and educational attainment. Equations of special situations for iterating the provincial population from one-year-old to “100+” with different educational attainments are shown as followings:

| If $edu\neq1, a=99$, | | |
| --- | --- | --- |
|  | ${Pm}_{yr,a+1,edu}=\left( {Pm}_{yr-1,a,edu}\times\left( 1-{MORm}_{yr,a,edu} \right)\times\left( 1+{NetPIM}_{yr,a,edu} \right)\times\left( 1-{Gm}_{yr,a,edu} \right)+{Pm}_{yr-1,a,edu-1}\times\left( 1-{MORm}_{yr,a,edu-1} \right)\times\left( 1+{NetPIM}_{yr,a,edu-1} \right)\times{Gm}_{yr,a,edu-1}+{Pm}_{yr-1,a+1,edu} \right)\times\left( 1+{NetGIM}_{yr} \right)$ | (S1a) |
|  | ${Pf}_{yr,a+1,edu}=\left( {Pf}_{yr-1,a,edu}\times\left( 1-{MORf}_{yr,a,edu} \right)\times\left( 1+{NetPIM}_{yr,a,edu} \right)\times\left( 1-{Gf}_{yr,a,edu} \right)+{Pf}_{yr-1,a,edu-1}\times\left( 1-{MORf}_{yr,a,edu-1} \right)\times\left( 1+{NetPIM}_{yr,a,edu-1} \right)\times{Gf}_{yr,a,edu-1}+{Pf}_{yr-1,a+1,edu} \right)\times\left( 1+{NetGIM}_{yr} \right)$ | (S1b) |
| If $edu=1, a\neq99$, | | |
|  | ${Pm}_{yr,a+1,edu}= {Pm}_{yr-1,a,edu}\times\left( 1-{MORm}_{yr,a,edu} \right)\times\left( 1+{NetPIM}_{yr,a,edu} \right)\times\left( 1-{Gm}_{yr,a,edu} \right)$ | (S2a) |
|  | ${Pf}_{yr,a+1,edu}= {Pf}_{yr-1,a,edu}\times\left( 1-{MORf}_{yr,a,edu} \right)\times\left( 1+{NetPIM}_{yr,a,edu} \right)\times\left( 1-{Gf}_{yr,a,edu} \right)$ | (S2b) |
| If $edu=1, a=99$, | | |
|  | ${Pm}_{r,yr,a+1,edu}=\left( {Pm}_{yr-1,a,edu}\times\left( 1-{MORm}_{yr,a,edu} \right)\times\left( 1+{NetPIM}_{yr,a,edu} \right)+{Pm}_{yr-1,a+1,edu} \right) \times\left( 1-{Gm}_{yr,a,edu} \right)$ | (S3a) |
|  | ${Pf}_{r,yr,a+1,edu}=\left( {Pf}_{yr-1,a,edu}\times\left( 1-{MORf}_{yr,a,edu} \right)\times\left( 1+{NetPIM}_{yr,a,edu} \right)+{Pf}_{yr-1,a+1,edu} \right) \times\left( 1-{Gf}_{yr,a,edu} \right)$ | (S3b) |

All variables and parameters have the same meaning as in the text. Equation S1a and S1b describe a special situation for population aged “100+” ($a+1=100$), which consider the population aged both 99 and “100+” in the previous year. Equation S2a and S2b demonstrate the special case for population of illiterate educational group ($edu=1$), which do not have an earlier education stage. Equation S3a and S3b are the situations for illiterate population aged “100+”.
